# A Rare Case of Valsartan-Induced Angioedema

**DOI:** 10.7759/cureus.85623

**Published:** 2025-06-09

**Authors:** Sandra Abadir, Harendra Ipalawatte, Jasprit Takher

**Affiliations:** 1 Internal Medicine, Los Robles Regional Medical Center, Thousand Oaks, USA

**Keywords:** anaphylaxis, angioedema, angiotensin ii receptor blocker, bradykinin, c1 esterase inhibitor, hypertension

## Abstract

Angioedema of the face and neck is a rare but potentially life-threatening condition induced by angiotensin-converting enzyme (ACE) inhibitors. While ACE inhibitors are commonly implicated, recent reports suggest angiotensin II receptor blockers (ARBs) may also trigger angioedema. This case report highlights the development of angioedema in a patient on valsartan, an ARB, emphasizing the importance of recognizing ARBs as a potential cause of this condition, even in patients with no prior history of swelling.

A 77-year-old male with coronary artery disease, hypertension, and type 2 diabetes presented to the emergency department with sudden-onset tongue swelling and difficulty breathing. The patient had been on valsartan for several years without any prior episodes of swelling. Upon examination, he had significant tongue swelling, and multiple hemorrhagic blisters were observed. Laboratory results indicated mild inflammation and C1 esterase inhibitor levels were normal, supporting a diagnosis of non-hereditary angioedema. Treatment included steroids, epinephrine, and diphenhydramine. Valsartan was discontinued and replaced with nifedipine. The patient improved, and the swelling resolved without airway obstruction.

This case underscores the need for heightened awareness of angioedema induced by ARBs like valsartan. Early recognition and discontinuation of the offending medication are key to successful management. In drug-induced angioedema without airway compromise, supportive care is typically sufficient, and the condition is self-limiting. Proper identification of the cause and timely intervention are crucial to avoid life-threatening complications.

## Introduction

Angioedema of the face and neck is a rare but potentially life-threatening condition that can arise as an adverse effect of angiotensin-converting enzyme (ACE) inhibitors [1]. The condition is characterized by spontaneous, asymmetric swelling of the deeper layers of the skin or mucosal membranes, typically involving the face, tongue, lips, and sometimes the upper airway [2]. Angioedema associated with ACE inhibitors has been well-documented in the literature and is recognized as a significant clinical concern, particularly due to its potential to cause life-threatening airway obstruction if left untreated [1].

Although ACE inhibitors are the most commonly implicated medications in the development of angioedema, recent reports have highlighted that angiotensin II receptor blockers (ARBs), which were initially designed to avoid the side effects of ACE inhibitors, including both angioedema and the persistent dry cough, may also be associated with this condition. Unlike ACE inhibitors, ARBs do not increase bradykinin levels in the serum, which was thought to be the primary mechanism behind ACE inhibitor-induced angioedema. However, increasing evidence suggests that elevated levels of angiotensin II can indirectly inhibit ACE activity, thus potentially triggering angioedema, even in patients taking ARBs [3].

The clinical manifestation of angioedema typically affects the head and neck, with the mouth, pharynx, and tongue being the most commonly involved areas. While the larynx is less frequently affected, its involvement poses a significant risk due to the potential for airway compromise [1]. Notably, angioedema associated with these medications is usually non-pruritic, differentiating it from other forms of allergic reactions that involve itching. It is estimated that angioedema occurs as a side effect of ARBs in approximately 0.11% of patients compared to 0.3% of patients on ACE-I [4]. The pathophysiological mechanisms involving increased angiotensin II levels and subsequent inhibition of ACE activity are thought to contribute to the development of this rare side effect [5].

Angioedema is generally classified into two types: hereditary and non-hereditary. Hereditary angioedema, a rare condition caused by a deficiency of C1 esterase inhibitor, typically presents with recurrent episodes of swelling beginning in childhood. The diagnosis is confirmed through measurement of low C4 and C1 esterase inhibitor levels. Non-hereditary angioedema can occur as a result of acquired C1 esterase inhibitor deficiency, which may be triggered by factors such as malignancy, drug reactions, or allergic responses to food or medications. Among the medications commonly implicated in non-hereditary angioedema, ACE inhibitors are the most frequently cited cause [1].

In this report, we present a case of angioedema induced by ARBs in a hypertensive patient who had been on valsartan for several years without any prior episodes of swelling. This case underscores the importance of considering ARBs as a potential cause of angioedema, even in patients without a history of such reactions, and highlights the need for heightened awareness of this rare but serious side effect.

## Case presentation

A 77-year-old man with a past medical history of coronary artery disease, hypertension, and type 2 diabetes mellitus was brought to the emergency department from his nursing home following a sudden onset of tongue swelling and difficulty breathing. The swelling developed rapidly, and the patient reported a sensation of tightness in his throat. He denied any recent food allergies, new food products, or changes in his diet. His medication regimen included valsartan 160 mg twice daily for hypertension and metformin 500 mg twice daily for diabetes, both of which he had been taking without any recent changes.

Upon admission, the patient was afebrile and normotensive, with a heart rate in the 70s and a respiratory rate between 14 and 17 breaths per minute. Oxygen saturation was stable at 96% on room air. Laboratory findings are included in Table 1. 

**Table 1:** Relevant laboratory findings along with normal values

Relevant Laboratory Test	Patient’s Value	Normal Value
Potassium	3.5 mmol/L	3.6-5.1 mmol/L
White blood cell	10.9x10³/µL	4.5-11.0x10³/µL
C-reactive protein	14.1 mg/L	<10 mg/L

Physical examination revealed significant tongue swelling, compromising the patient's airway. Multiple blisters with active hemorrhaging were observed underneath the tongue, but the patient did not appear to be in acute respiratory distress. He demonstrated no increased work of breathing, and there was no evidence of other signs of airway obstruction, such as stridor or hoarseness. Despite the severe presentation, he remained stable maintaining adequate oxygenation.

Given the rapid onset of symptoms and the patient’s medication history, a diagnosis of valsartan-induced angioedema was considered. The patient was treated promptly with intravenous dexamethasone 10 mg, epinephrine 1 mg, and diphenhydramine 25 mg to address the swelling and prevent further airway compromise. He was admitted to the intensive care unit (ICU) for close monitoring and was kept nothing by mouth (NPO). In response to the suspected drug-induced angioedema, his valsartan was discontinued and replaced with slow-release nifedipine for hypertension management.

The patient was also started on intravenous methylprednisolone 60 mg every six hours, which was later switched to oral prednisone 40 mg daily. After monitoring the patient in the ICU for 24 hours during the treatment period, his condition improved, and the swelling began to resolve without progression to airway obstruction. C1 esterase inhibitor levels, which resulted prior to discharge, were found to be 18 mg/dL (normal range: 16-33 mg/dL), further supporting the diagnosis of non-hereditary angioedema rather than hereditary causes.

The patient was discharged once he was medically stable, with instructions to avoid any ACE inhibitors or ARBs in the future. He was also advised to follow up with his primary care physician and cardiologist for ongoing management of his hypertension and diabetes.

## Discussion

Angioedema is a non-pitting, asymmetric swelling primarily affecting the face, throat, and extremities. It results from the extravasation of local plasma into the interstitial space, typically developing rapidly within minutes and resolving within 24 to 48 hours in most cases [6]. This condition can be spontaneous or triggered by various factors, including medications, infections, and allergens. Angioedema has been recognized for centuries, with the earliest descriptions dating back to the 1500s [7].

The pathophysiology of hereditary angioedema, a rare but well-defined form, began to be understood in the 19th century when a family presented with recurrent episodes of what was described as "fatal suffocation," a phenomenon later attributed to hereditary angioedema. In 1963, the underlying cause was identified as a deficiency of C1 esterase inhibitor, a protein that regulates the complement and kinin systems. The deficiency of this inhibitor leads to excessive bradykinin production, which results in the characteristic swelling of the skin and mucosal tissues [7,8].

Bradykinin is also believed to play a central role in the development of angioedema induced by ACE inhibitors. ACE is similar to kininase II, an enzyme, responsible for metabolizing and inactivating bradykinin. By inhibiting ACE, ACE inhibitors increase the accumulation of bradykinin in the plasma, which contributes to the development of angioedema [9]. To mitigate this risk, ARBs were developed in the 1990s. Unlike ACE inhibitors, ARBs, a class of medication that has been used to lower blood pressure and reduce proteinuria, left ventricular hypertrophy, and improve outcomes in heart failure, do not affect bradykinin levels but instead block the action of angiotensin II [10]. 

Angioedema can be classified into four categories: (1) hereditary angioedema, an autosomal dominant condition caused by C1 esterase inhibitor deficiency, characterized by recurrent attacks beginning in childhood and worsening during puberty; (2) mast cell-mediated angioedema, which occurs when IgE binds to mast cells, triggering degranulation and leading to symptoms such as pruritus, urticaria, hypotension, and anaphylaxis; (3) kinin-related angioedema, which results from overproduction of bradykinin or inhibition of its degradation, most commonly due to ACE inhibitors; and (4) angioedema of unknown etiology, typically a chronic condition that is diagnosed when patients experience three or more episodes within a year, often accompanied by pruritus and urticaria [7].

Angioedema, particularly induced by ACE inhibitors and ARBs, remains a significant clinical concern due to the potential for life-threatening airway compromise. Our case of valsartan-induced angioedema in a 77-year-old hypertensive patient underscores the importance of considering ARBs as a cause of this condition, even in patients without a prior history of swelling. Although ACE inhibitors are the most commonly cited cause, recent reports have demonstrated that ARBs, including valsartan, can also lead to similar adverse reactions, albeit less frequently. In contrast to ACE inhibitors, which increase bradykinin levels, ARBs were thought to avoid this issue, as they do not influence bradykinin metabolism. However, as highlighted by Makani et al. [4], ARBs can indirectly elevate angiotensin II levels, which may subsequently inhibit ACE activity and trigger bradykinin-mediated angioedema. Our case supports this hypothesis, as the patient's symptoms of tongue swelling, without pruritus or systemic allergic manifestations, align with the presentation of bradykinin-induced angioedema. This is consistent with previous literature, where ARB-induced angioedema was noted to occur in approximately 0.11% of patients, much less frequently than with ACE inhibitors, yet still poses a significant risk in susceptible individuals [4].

A unique feature of our case was the absence of prior episodes of swelling in the patient despite long-term use of valsartan. This highlights a critical aspect of ARB-induced angioedema: while it is rare, it can occur unexpectedly, even in patients with prolonged exposure to the drug. Most previous studies emphasize that drug-induced angioedema typically presents shortly after initiating treatment, or after a dosage change, yet our patient had been on valsartan for years without previous issues. This emphasizes the need for clinicians to be aware of the possibility of delayed-onset angioedema, even in patients with long-term stable use of ARBs [1].

In our case, the patient presented with angioedema without urticaria, systemic manifestations, or prior episodes, and had normal C1 esterase inhibitor levels. This presentation, coupled with the patient’s use of valsartan, points to kinin-related angioedema as the most likely diagnosis.

Management of angioedema depends on the underlying cause and the severity of symptoms. The first priority is to assess and secure the airway, as patients with tongue swelling, laryngeal edema, or stridor may require endotracheal intubation. If the angioedema is histamine-mediated, such as in an allergic reaction, epinephrine, diphenhydramine, and corticosteroids are appropriate treatments. For patients with hereditary angioedema due to C1 esterase inhibitor deficiency, fresh frozen plasma may be required. Importantly, if the angioedema is induced by ACE inhibitors or ARBs, discontinuation of the offending drug is crucial [7].

Comparing the treatment approaches in our case to those in the literature, we used a combination of corticosteroids (dexamethasone then methylprednisolone), epinephrine, and antihistamines to manage the swelling and prevent airway compromise. This is consistent with general treatment protocols for drug-induced angioedema, which emphasize airway management and anti-inflammatory therapy. However, while most cases of ACE-inhibitor-induced angioedema resolve upon discontinuation of the offending drug and use of corticosteroids, the use of epinephrine in our case was particularly significant given the rapid onset of tongue swelling and the potential for airway obstruction. In contrast, some reports have emphasized the use of fresh frozen plasma in hereditary cases of angioedema due to C1 esterase inhibitor deficiency [7], which was unnecessary in our patient due to normal C1 esterase levels.

## Conclusions

ARB-induced angioedema remains less common compared to ACE inhibitor-induced cases but is still a serious condition that can present unexpectedly, even after years of stable medication use. This case underscores the need for clinicians to remain vigilant for signs of delayed-onset angioedema, as early recognition and prompt treatment, including the use of corticosteroids, antihistamines, and epinephrine when necessary, are critical for preventing airway compromise and ensuring favorable outcomes. Our findings support the growing body of literature that highlights ARBs as a potential cause of drug-induced angioedema, warranting careful consideration in the management of patients on these medications.
